# Practical scale modification of oleogels by ultrasonic standing waves

**DOI:** 10.1016/j.ultsonch.2022.105970

**Published:** 2022-03-03

**Authors:** Petri Lassila, Fabio Valoppi, Oskari Tommiska, Jere Hyvönen, Axi Holmström, Sami Hietala, Ari Salmi, Edward Haeggström

**Affiliations:** a Electronics Research Laboratory, Department of Physics, University of Helsinki, P.O. Box 64 (Gustaf Hällströmin katu 2), FI-00014, Finland; b Department of Food and Nutrition, University of Helsinki, P.O. Box 66 (Agnes Sjöbergin katu 2), FI-00014, Finland; c Helsinki Institute of Sustainability Science, Faculty of Agriculture and Forestry, University of Helsinki, FI-00014, Finland; d Department of Chemistry, University of Helsinki, P.O. Box 55 (Virtasen aukio 1), FI-00014, Finland

**Keywords:** Oleogels, Ultrasonic standing waves, Treatment chamber design and development, Mechanical properties, Hyperelastic foam, Oil release

## Abstract

Lipid-based materials, such as substitutes for saturated fats (oleogels) structurally modified with ultrasonic standing waves (USW), have been developed by our group. To enable their potential application in food products, pharmaceuticals, and cosmetics, practical and economical production methods are needed. Here, we report scale-up of our procedure of structurally modifying oleogels via the use of USW by a factor of 200 compared to our previous microfluidic chamber. To this end, we compared three different USW chamber prototypes through finite element simulations (FEM) and experimental work. Imaging of the internal structure of USW-treated oleogels was used as feedback for successful development of chambers, *i.e.,* the formation of band-like structures was the guiding factor in chamber development. We then studied the bulk mechanical properties by a uniaxial compression test of the sonicated oleogels obtained with the most promising USW chamber, and sampled local mechanical properties using scanning acoustic microscopy. The results were interpreted using a hyperelastic foam model. The stability of the sonicated oleogels was compared to control samples using automated image analysis oil-release tests. This work enabled the effective mechanical-structural manipulation of oleogels in volumes of 10–100 mL, thus paving the way for USW treatments of large-scale lipid-based materials.

## Highlights

• 10–100 mL ultrasonic standing wave chamber with an acoustically soft reflecting boundary for oleogel structural modification was developed.
• Bulk and local mechanical properties of modified oleogels were studied by uniaxial compression tests and by scanning acoustic microscopy.
• Structural properties of oleogels were elucidated by fitting a hyperelastic foam model on uniaxial compression data.
• The stability of modified oleogels was compared to control samples using an automated image analysis oil-release method.

## 1 Introduction

The food, cosmetic, and pharma industries largely use semi-solid lipid materials because of their texture, functionality, and ability to deliver liposoluble molecules[1], [2]. These lipids can be extracted from living organisms, or obtained through hydrogenation of liquid oils[3], [4], [5]. Solid fats produced by these methods often result in high concentrations of saturated fats, whose excessive consumption has been shown to positively correlate with cardiovascular diseases, type 2 diabetes, metabolic syndrome, and eventual onset of obesity[6], [7]. Due to these adverse health effects, the development of semi-solid lipid-based materials through physical methods was initiated two decades ago[8]. Within this field, fat mimetics obtained through structuration and gelation of emulsions and gelation of oils (oleogels) present a reduced concentration of saturated fats, thus forming healthier lipid materials[2], [9], [10].

Oleogels are viscoelastic and self-supporting materials that largely consist of liquid oil gelled using structuring agents, such as polysaccharides, proteins, or low molecular weight self-assembling molecules *e.g.,* monoglycerides, fatty alcohols, fatty acids, waxes, or phytosterols and their esters[2], [10]. Oleogels can be produced through direct and indirect methods[2]. The direct method consists of a thermal cycle applied to a mixture of oil and structuring agents. During cooling, structuring agents experience first- or second-order phase transitions, *i.e.,* crystallization or glass transition, respectively, eventually self-assembling into a network that traps the oil[2], [11], [12]. Indirect methods use oil-insoluble molecules and require additional solvents (*e.g.,* water, acetone, ethanol, tetrahydrofuran), which are then removed from the system using drying methods, or substituted stepwise with oil[9]. Indirect methods comprise solvent exchange, emulsion-, foam-, and aerogel-templated methods. Between these two methods, the direct method is more relevant to industry and is exploitable, due to its celerity and convenience[13].

However, oleogels still have problems with storage and stability and are structurally weak, which can impede their application in products[8]. Indeed, oxidation of the oil fraction worsens the nutritional profile of oleogels, rearrangement of the crystalline network during storage can lead to release of oil from the oleogel, and large deformations (such as mixing, spreading, and lamination) can irreversibly damage the oleogel structure, softening the material and leading to oil release[14], [15], [16]. Engineering of oleogels by modifying formulation and processing parameters can overcome these limitations. Some examples of the main strategies used to counteract issues present in oleogels include use of antioxidants[17], (ii) blending different oils[18], (iii) changing the ratio between structuring agents and oil[19], (iv) using mixtures of structuring agents[20], [21], (v) using an inert atmosphere[17], (vi) modulating thermal cycle parameters (heating and cooling rate, isothermal holding time)[15], [22], (vii) application of shear via a laminar shear crystallizer[23], (viii) and application of high-intensity ultrasound[24], [25].

Unfortunately, none of these strategies provide the means to modify the oleogel nano- and microstructure in a precise and controlled manner, which could possibly ameliorate oleogel stability as proven for saturated fats[26]. Such control may be obtained through the application of ultrasonic standing waves (USW) during oleogel formation, as we recently demonstrated through mathematical modelling, computer simulations, and experimental work[27], [28]. This new oleogel structure consisted of alternated bands of higher- and lower-density structuring agent microcrystalline aggregates[28]. USWs present localized pressure gradients where stacked planes of high-pressure (antinodes) and low-pressure (nodes) zones exist[29]. Pressure gradients exert an acoustophoretic force on solid particles present in the field, moving them towards the nodes due to their higher density compared to the medium where they are suspended[30], [31], [32]. The shape of the standing wave field gives rise to the band-like structure in oleogels, which can be tailored through manipulation of the frequency of the transmitted wave. This new oleogel structure can be achieved regardless of the type and concentration of the crystallizing structuring agent and oleogel cooling rate[28].

Our previous work was based on a microfluidic batch chamber, where we demonstrated the feasibility of oleogel structure manipulation with USWs[27], [28]. However, the design of the microfluidic chamber made sample extraction impractical because of irreversible damage to the oleogel and only allowed us to perform limited characterization through optical microscopy and X-ray diffraction analysis[28]. Here, we developed three new USW chambers, with volumes from 10 to 100 mL, which were able to form a controllable standing field during oleogel formation and allowed sample extraction. USW-treated oleogels were then analysed through optical and ultrasound imaging, uniaxial compression, and oil release. FEM simulations were employed to obtain insights on the USW shape. Mechanical analysis and oil-release results were fit with a hyperelastic foam model and a power law model, respectively, which allowed further understanding of USW effects on oleogel structure. This practical method possibly enables the use of USW-treated lipid-based materials in foods, pharmaceuticals, and cosmetics.

## 2 Materials and methods

### 2.1 Materials

Rapeseed oil and plastic film (cling film) were purchased from a local grocery store. Myverol™ saturated monoglyceride (MG) (fatty acid composition 1.4% C14:0, 59.8% C16:0, 38.8% C18:0; melting point 68.0 ± 0.5 °C) was donated by Kerry Ingredients and Flavour (Bristol, United Kingdom). Acrylonitrile butadiene styrene (ABS) 3D printer material was purchased from Ultimaker B.V. (Utrecht, The Netherlands). Piezoelectric ceramics (Pz26, with nominal resonances of 2 MHz) were purchased from Meggit Ferroperm Piezoceramics (Kvistgaard, Denmark). Transparent plastic printer film was acquired from Folex Imaging (Seewen, Switzerland).

### 2.2 Experimental chamber

Oleogels were prepared by adding 10% or 5% (w/w) MG to rapeseed oil at 80 °C under magnetic stirring. Samples were mixed for at least 15 min after the crystalline material was molten. Samples were next transferred to the experimental chamber. We compared the following three different designs: a direct upscale of the microfluidic chamber concept, featuring an ultrasonic transducer and a glass reflector (hard boundary); a scaled-up microfluidic chamber concept with a soft reflective boundary (liquid-air boundary, no reflector); and a chamber featuring a liquid matching layer between the transducer and the sample. The experimental chambers were designed with the open-source 3D computer graphics software Blender (Blender foundation) and printed with ABS using the 3D printer Ultimaker 3. The top reflector, used in the first chamber design, was a glass Petri dish with a diameter of 54 mm attached to a 3D printed connector, which in turn was mounted on a vertical translation stage (KM200B/M, Thorlabs, Newton, NJ, USA). A plastic film was coupled with Aquasonic 100 ultrasound transmission gel (Parker Laboratories, Inc., Fairfield, NJ, USA) to the reflector surface to enable removal of the reflector from the crystallized oleogel. The sonicating transducer was connected to a PicoScope 2000 series oscilloscope (Pico Technology, Cambridgeshire, United Kingdom), a HV pulser/receiver (Olympus Corporation, Shinjuku, Tokyo, Japan) and a RF power amplifier (Tomco Technologies, Stepney SA, Australia). The pulser was disconnected when sonicating. The sonication waveform was controlled with an arbitrary waveform generator (Hewlett Packard, Palo Alto, California, USA).

Transparent printer film (Folex Imaging, Seewen, Switzerland) was used in the third chamber design to construct the inner and outer walls of the chamber by cutting sufficiently long strips and wrapping them around the 3D printed body. Cling film was tightly fastened between the inner and outer cylindrical walls such that the opening of the inner cylinder was covered by the film, effectively separating the chamber into a lower and upper part. The walls were made watertight with superglue (The Gorilla Glue Company, Sharonville, Ohio, USA), parafilm (Bemis Manufacturing Company, Sheboygan Falls, WI, USA), and clear packing tape (3 M, Saint Paul, MN, USA). Chamber designs were attached to a commercial tilt stage (KM200B/M, Thorlabs, Newton, NJ, USA) and tightly fastened by hand via a nut implanted in a 3D-printed housing. The sonicating transducer was monitored using the oscilloscope. For the reflector case, maximum amplitude was obtained when the distance (*d*) between the transducer and the top reflector satisfied Eq. (1):

(1) $$d=n\frac{\lambda }{4}\left(n=2,4,6,8\cdots \right)$$

where *λ* is the wavelength of the ultrasound in oil, and *n* is an integer corresponding to twice the number of nodal or anti-nodal planes in the experimental chamber.

A layer of rapeseed oil (30 mL, 10 mm thick) was placed in the lower chamber, which was then preheated by driving the piezo at high power. The molten oleogel was then transferred to the upper chamber. In the reflector case, the reflector was immediately lowered and aligned with the tilt stage and pulser. In the open-top case, the molten oleogel surface and the piezo were aligned using the same method. The amplitude of the drive signal was limited to a range where no adverse streaming or excessive heating occurred (8–24 V_pp_). The transmitting frequency was selected by measuring the resonance frequency of the transducers using a vector network analyser (NanoVNA by edy555) and was determined to be 2.25 MHz. An experiment to determine any potential temperature dependence of the piezo resonance frequency was performed; no significant change was observed. The transmitting frequency was kept constant throughout the sonication. The sonication continued until the sample had crystallized (after the oleogel had turned opaque). Reference samples were prepared in the same way, without applying sonication. The cooling rate was between 3.8 °C/min and 1.3 °C/min for chamber #3 (for more information see Fig. S2), which was measured using a Pt100 resistance temperature detector (NB-PTCO-187, TE Connectivity, Switzerland) with a 24-bit analog-to-digital converter (ADS1220, Texas Instruments, TX, USA) in a ratiometric four-wire configuration with a low side reference. The Pt100 was embedded in epoxy resin to provide electrical insulation and mechanical support. Two 3.09 kΩ (±0.1%) thin-film precision resistors (RN73C1J3K09BTDF, TE Connectivity, Switzerland) stacked parallel were used as reference resistance. The acquisition was controlled with an ESP32 microcontroller (Espressif, China) with custom firmware (written in C/C++), which also relayed the data wirelessly for storage and handling in a TIG-Stack (Telegraf-InfluxDB-Grafana) running on a virtual server along with an MQTT Broker (Eclipse MosQuitto)[33].

After overnight annealing, crystallized oleogels were cut with a thin-walled cylinder (diameter 20 mm, wall thickness 0.3 mm) made from aluminium or copper. To remove the oleogel from the cylinder, air was blown into the open end of the cylinder such that the sample slid out without damaging itself. Slices from the sample disk were cut with a microscope glass cover slip (Menzel-Gläser, Braunschweig, Germany), such that the cut slice remained on the cover slip after the cut. Another cover slip was then placed on the other side of the slice such that the sample was sandwiched between two microscope cover slips. The sample was propped upright and pictures of the slice were taken with a digital microscope (Q-scope model QS.80200-P, Euromex Microscopen BV, Arnhem, The Netherlands).

### 2.3 Simulation and fitting methods

Simulations were performed using a finite-element-method (FEM) simulation software, COMSOL Multiphysics 5.6 (Comsol Inc., Sweden). Simulation models employed Pressure Acoustics, Solid Mechanics, and Electrostatics physics interfaces. All couplings between individual physics interfaces were performed using COMSOL’s built-in Multiphysics-interfaces. The Pressure Acoustics interface was used to model acoustic waves in the oil and oleogel domains. The Solid Mechanics interface was used to model elastic waves in the solid parts of the chambers along with the piezoelectric effect driving the Pz26 piezoceramic disk (when coupled with the Electrostatics interface).

**Fig. 1:**
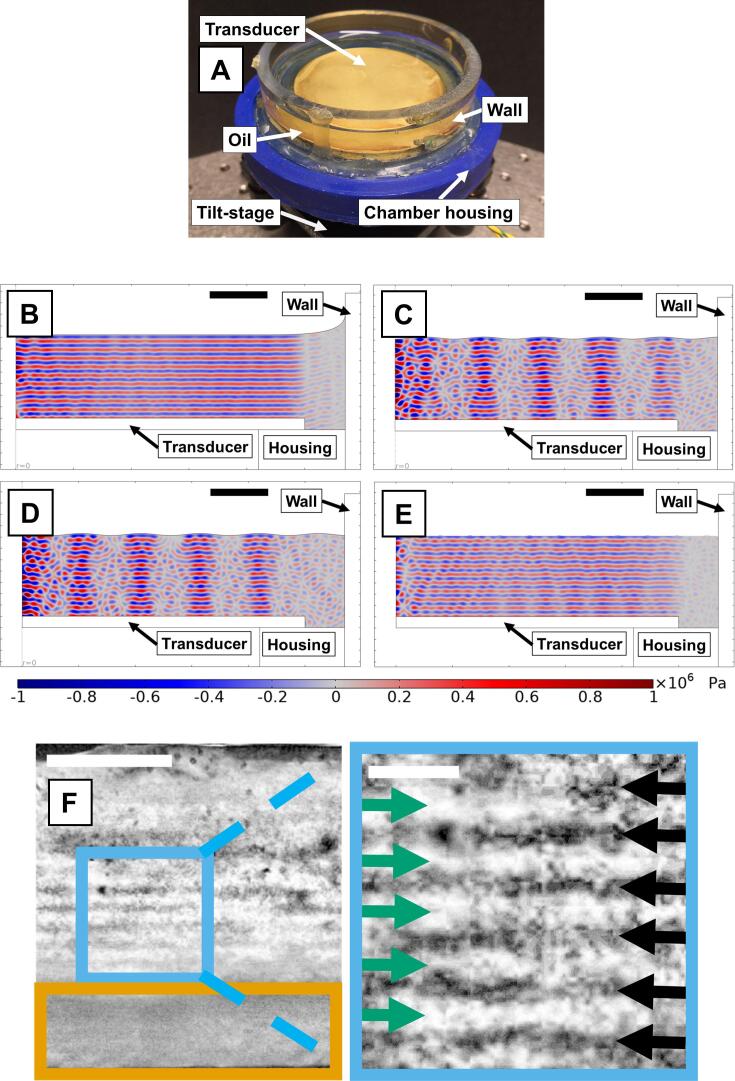
(A) Second design of the scaled-up chamber. (B) Simulation of the second chamber design with a meniscus contained to the edge, *i.e.* the surface over the transducer is mostly flat. (C and D) Simulations of the second chamber design with an oscillating oil surface where (C, arrow) crest and (D, arrow) through height are λ/4. (E) Time-averaged field inside the second chamber design with an oscillating surface. (F) 10% monoglycerides containing sample sonicated with the second chamber design. Green and black arrows indicate regions of lower and higher concentration of MG oleogelator, as interpreted from the darker and lighter shade. Size bar represents 2 mm on the original picture and 0.5 mm in the zoom in. (B-E) Size bars equal 5 mm.

The piezo element was driven with 10 V_pp_. Rapeseed oil was modelled with the following material properties: sound speed 1480 m/s, density 0.9073 g/cm^3^, dynamic viscosity 0.03 Pa*s, and bulk viscosity 0.02 Pa*s. The oleogel (considered as molten oleogel) had the following material properties: sound speed 1480 m/s, density 0.92 g/cm^3^, dynamic viscosity 0.015 Pa*s, bulk viscosity 0.01 Pa*s. All simulation geometries were 2D-axisymmetric. Chamber #2 geometry consisted of a piezoceramic disk (diameter 5 cm), a plastic holder for the piezo (polycarbonate), an oil domain, and a plastic container enclosing it (PMMA). The depth of the oil domain was 9.75 times the acoustic wavelength in oil. The curved oil surface (Fig. 1B and Fig. S3B) was approximated with the curve drawing tool. The periodic oil surface was built using the parametric curve drawing tool. The design geometry (two layers) of chamber #3 (Fig. S6B) had a similar structure to the design of chamber #2, but a thin (10 µm) PVC plastic sheet and a fluid domain (molten oleogel) was placed on top of the oil domain. The depth of the oil domain was 13 times the wavelength and the depth of the oleogel domain was 13.1 times the wavelength. To reduce the size of the model domain, the outer container wall was approximated as a hard wall boundary condition. The simulation model for chamber #2 did not feature a plastic film between the transducer and molten oleogel.

The sample material properties were modelled using a hyperelastic material model, which can be used to model large strains in organic and rubber-like materials. When using the Storåker’s hyperelastic model, the first Piola-Kirchhoff stress during uniaxial compression or tension can be expressed as the following:[34]

(2) $$P=\sum_{i=1}^{k}\left(2\frac{\mu_{i}}{\alpha_{i}}\left(\lambda^{-\alpha_{i}\frac{1+3\beta_{i}}{1+2\beta_{i}}}-1\right)\lambda^{\alpha_{i}-1}\right)$$

where *λ* is the stretch of the sample and parameters *µ*, *α*, and *β* describe the material properties of the sample. The *α* parameter has no intrinsic physical meaning[35]. The *µ* parameter represents the shear modulus of the material and the *β* parameter is related to the Poisson’s ratio (*ν*) through Eq. (3)

(3) $$\nu =\frac{\beta }{1+2\beta }$$

The material parameters for both the reference sample and the sonicated sample were obtained by fitting Storåker’s model to the measurement data from the compression experiment. Fitting was performed with a custom fitting tool written in MATLAB and was limited to stretches in the range [0.65, 1]. During plotting, we used strain instead of stretch, since the fitted material model did not accurately follow the experimental data below 0.65 stretch. To select *k* for the fitted material model, Akaike Information Criterion (AIC) was employed[36]. AIC was used to evaluate fits performed with *k* = 1, 2, and 3 to the 5% and 10% reference samples. The residual sum of squares (RSS, see Eq. (4)) was used to determine goodness-of-fit during each iteration of curve fitting. As the fitting problem was nonlinear and likely to contain multiple local minima, a *GlobalSearch* function with *fmincon* solver was used to find the global minimum of the quality function in the range of parameter values deemed physical.

(4) $$\mathrm{RSS}=\sum_{i=1}^{n}{\left(y_{i}-\hat{y_{i}}\right)}^{2}$$

where *y_i_* is the value to be predicted and *ŷ_i_* is the predicted value of *y_i_*. The model function was used to calculate the values of *ŷ_i_* for the quality function.

After solving values for material parameters, the standard errors of individual parameters were calculated. Errors were estimated from the variance-covariance matrix, obtained by inverting the Hessian matrix obtained from the solver. As the Hessian matrix calculated by MATLAB’s *fmincon* function can be inaccurate, the fitting was redone with the *fminunc* function, using the earlier solved values as the starting point and the Hessian matrix of the output. Material parameters obtained from fitting were then imported to the COMSOL model.

### 2.4 Ultrasound microscopy for determination of local mechanical properties

A Coded Excitation Scanning Acoustic Microscope (CESAM) built previously[37] was used to determine local acoustic attenuation in sliced oleogel samples. The device employs coded excitation, which increases the signal-to-noise ratio of the measurement (when compared to conventional excitation), thus enabling performance of large-area pulse-echo measurements through the sample without any averaging. For this application, we used a spherically focusing 30-MHz immersion transducer (Panametrics V375, 30/25, F = 0.75″ OLF, from Baker Hughes, Houston, TX, USA). The frequency range (8–30 MHz) was chosen as a compromise between frequency-dependent attenuation, lateral resolution, and axial resolution (signal bandwidth).

Several 2-mm thick samples were prepared with the method described in the final paragraph of the Experimental Chamber section (section 6.2) and were then placed on 200-µm thick 22x22 mm glass microscope slides. No second cover slip was used, and one face of the gel remained uncovered. The sample was mapped with a 2D raster scanned grid. Reference signals were acquired from scanned areas with only a water-glass interface, while sample signals were acquired from areas where the sample was present. A normalized frequency spectrum was obtained by taking the Fourier transform of the reference and sample signal, and dividing the Fourier transformed reference signal with the Fourier transformed sample signal[38].

A least-squares power-law fit (Eq. (5)) was fitted to the normalized spectrum and the attenuation coefficient γ and exponent *y* were extracted.

(5) $$\frac{\ln N\left(\omega \right)}{\Delta x}=\gamma \omega^{y}$$

where $N\left(\omega \right)$ is the normalized spectrum, Δx is the travel distance of the propagating wave, *γ* is the attenuation coefficient, ω the angular frequency, and *y* is the frequency-dependent exponent. The loss tangent can be calculated for acoustic waves from Eq. (6) [39].

(6) $$\tan\left(\delta \right)=\frac{M^{\text{'}\text{'}}}{M^{\text{'}}}=\frac{2\alpha c}{\omega }=2\omega^{y-1}\gamma c$$

where *M''* and *M'* are the imaginary and real components of the complex longitudinal wave modulus, respectively, α is attenuation which depends on *γ* and *ω*^y^, and c is the phase velocity.

### 2.5 Mechanical analysis

Samples for mechanical testing were produced with the method described in the experimental chamber section (section 6.2) with the third chamber design. Testing was performed on a Discovery Hybrid Rheometer 2 (TA-instruments, New Castle DE, USA). In uniaxial compression, measurements were performed using a 40-mm parallel plate geometry. Compression was performed by moving from a gap of 10 mm to 3 mm at a constant linear rate of 5 µm/s. Measurements were performed at 22 °C. Data were collected with Trios software version 4.1 (TA Instruments) and exported as text files for analysis in MATLAB R2020. Data points belonging to each replicate of the same sample treatment were interpolated from the experimental data with a spline function to produce error bars.

### 2.6 Oil-release test

Oil-release tests were conducted as previously described[40]. Oleogels were cut in cylindrical shape (diameter 20 mm) and placed on Whatman® qualitative filter paper, Grade 2 (General Electric, Fairfield, CT, USA). The release process was imaged every 10 min for 12–24 h using a Lenovo Essential FHD Webcam (Lenovo Group Limited, Peking, China) placed under the filter paper. The filter paper was placed 14.4 cm from the camera, on top of a circular laboratory tripod (height 20.5 cm, outer diameter 15 cm, inner diameter 12 cm). Non-linear regression analysis was performed using Eq. (7):

(7) $$A=K\cdot t^{n}$$

where *A* is the total area of the oil stain, *K* is the oil spread constant, *t* is time, and *n* is the exponent of the function. The Trust-Region-Reflective-Algorithm was used to performed non-linear least-squares function minimization, and the goodness of fit was evaluated based on statistical parameters of fitting (R^2^, p, standard error) and the residual analysis.

## 3 Results & discussion

### 3.1 Experimental chamber development

Oleogels are shear-sensitive materials, which undergo extensive structural damage upon mixing, spreading, and scooping[41]. We demonstrated in our previous work that by using USWs, it is possible to tailor the crystalline microstructure of oleogels to potentially enhance their physical stability[27], [28]. However, the microfluidic chamber we previously developed (Fig. S1A) for modifying oleogel structure had sample extraction and volume limitations, which prevented thorough characterization of the sonicated oleogels. In this work, we overcame these limitations by developing new experimental chambers. The new setups were designed with common features that enable further study of the mechanical properties and oil-release rate of ultrasonically modified oleogels. These features were larger process volumes (10–100 mL), ability to withdraw the sample with little or no damage, and no contact between the sample and the ultrasonic transducer.

The first design was based on our previous work[28] (Fig. S1B). The chamber consisted of a tilt stage mounting a 3D printed housing with detachable transparent chamber walls, onto which a piezoelectric transducer was mounted. A glass Petri dish was attached to a separate translation stage and its outer surface was used as an acoustic reflector. Thin plastic films were coupled to the transducer and reflector surfaces with ultrasonic water-based gel to enable post-sonication sample extraction. The translation stage permitted precise control of the distance between the transducer and reflector, which allowed selection of the number of nodal planes in accordance with Eq. (1). The whole setup was fixed on an optical table. To form a standing wave, the transducer and reflector first need to be aligned in parallel. To this aim, after pouring oil or molten oleogel into the chamber, the reflector was positioned above the transducer. Subsequently, a series of acoustic pulses were emitted, and their echoes were recorded with the transducer. Alignment was achieved by tilting the transducer with the tilt stage and was considered attained when the intensities of the echoes were maximized. An example of aligned and misaligned setups is shown in Fig. S2A and Fig. S2B. To determine the formation of a standing wave, we sonicated a dispersion of carbon rods (length 30–175 µm) in oil. The sonication parameters were selected based on our previous work[28] and preliminary experiments. We observed the formation of a band-like structure, where carbon rods were stacked in parallel planes (data not shown). As discussed in our previous work, this confirms the presence of a standing wave[27], [28]. The same procedure was applied during the crystallization of 10% monoglyceride-containing (MG) oleogels. Oleogels containing 4–6% MG are usually studied for saturated fat replacement in food products[25], [42]. However, we selected this MG concentration for its higher crystalline content, which produces oleogels more resistant to mechanical stress[25], [43]; this reduces complications during oleogel extraction. However, we did not exceed 10% MG concentration to avoid issues related to the dense crystalline network, where the crystalline organization may be difficult to determine[28]. After treatment and overnight annealing, oleogel samples were extracted from the chamber. Although we used a plastic film coupled to the transducer and the reflector, sample extraction always led to irreversible structural damage, making the sample unusable. To solve this problem related to sample extraction, we removed the glass reflector, introducing a soft reflective boundary (liquid/air). The setup for the second chamber is shown in Fig. 1A.

It is known that a phase-flip is introduced in the reflected wave when moving from an acoustically hard boundary to a soft one. This flip introduces a shift of λ/4 in the standing wave, resulting in the swap between the position of the nodes and anti-nodes. Formally, this is expressed with n assuming odd values in the boundary condition equation for standing waves (Eq. (1)). However, the overall shape of the standing wave and the resulting pressure gradients are not modified by the phase-flip[27]. The structural modification introduced by sonication of the oleogel is expected to remain identical. In this case, to generate a standing wave, the transducer also needs to be parallel to the reflecting boundary. However, the liquid surface can form a meniscus, which can disturb the formation of a standing wave. To understand the effect of the meniscus, we performed FEM simulations on our chamber geometry (Fig. 1B). The simulations highlighted that if the meniscus is confined to the edges of the chamber, *i.e.,* the curvature of the surface is mainly outside the area of the radiation from the piezoelectric ceramic, and there is a relatively flat surface on top of the piezo, the standing wave formation is not disturbed (*cf*. Fig. 1B, with Fig. S3A and Fig. S3B).

Operation of the chamber using a soft boundary configuration produced visible oscillations (ripples) in the oil surface along the z-direction (Video 1, supplementary materials). The oscillations were in the range of some Hz with an approximate amplitude of λ/4. Although ripples could have disturbed the formation of a standing wave, we still observed the formation of a band-like structure in a dispersion of carbon rods in oil. To understand this behaviour, we performed FEM simulations of a periodically oscillating soft boundary (simplified as a standing wave where the surface is displaced normal to the transducer surface) and its effect on the resulting waveform generated by the transducer. We considered three different surface displacement amplitudes (λ/4, λ/2, λ) at two spatial frequencies (number of crests over length 0.2 mm^−1^ and 1.2 mm^−1^) (Fig. 1C, Fig. 1D, and Fig. S3C to Fig. S3F). Simulations highlighted that for the spatial frequency 0.2 mm^−1^ and surface displacement amplitude of λ/4 (similar to the experimental observations), the area under the surface crest shows the formation of a standing wave, whereas the area under the valley shows a disrupted waveform (Fig. 1C and Fig. 1D). Being an oscillating surface, the crests and valleys are switching places, leading to an alternating presence of the standing wave over time (cf. Fig. 1C and Fig. 1D). This is because the disrupted waveform under the valleys has a lower-pressure amplitude compared to that of the standing wave formed under the crests. A full animation is provided in the supplementary material (Animation 1, supplementary material). During oleogel formation, monoglyceride crystals move to the nodes in a few seconds to minutes[28], and since we estimated that the frequency of the surface oscillations is 5 Hz, the crystals will experience the time-averaged wave (Fig. 1E), leading to the formation of a band-like structure over time. By increasing the surface displacement amplitude (λ/2, λ), the simulations show a disrupted field for all amplitudes at maximum displacement (Fig. S3C and Fig. S3D), possibly reducing the effect of sonication on oleogel microstructure band formation. On the other hand, a spatial frequency of 1.2 mm^−1^ leads to disrupted waveforms (Fig. S3E and Fig. S3F) regardless of the surface displacement amplitude. In conclusion, the simulations highlighted that even though a flat boundary is still the ideal case, small deformations of the surface still allow the formation of a time-averaged standing wave.

Next, we treated 10% MG oleogels during crystallization. After annealing, oleogels were easily extracted with little or no damage. After sectioning, the structure of the oleogels appeared inhomogeneous (Fig. 1F). Parallel dark and light bands were optically observed in the part of the sample close to the soft boundary (green and black arrows, Fig. 1F). As previously discussed[28], these bands correspond to optically denser and sparser monoglyceride crystalline structures. However, crystalline bands were absent in the area closest to the transducer (Fig. 1F, orange box). Transducers generate near-field effects, which were calculated to exist within 1 mm from the transducer surface (see calculations in supplementary material). These effects can propagate further than the near-field, disturbing the pressure gradient formed between nodal- and anti-nodal planes, possibly generating streaming[44], [45], [46] and leading to oleogel structure disruption within 2 mm from the transducer surface (Fig. 1F, orange box).

**Fig. 2:**
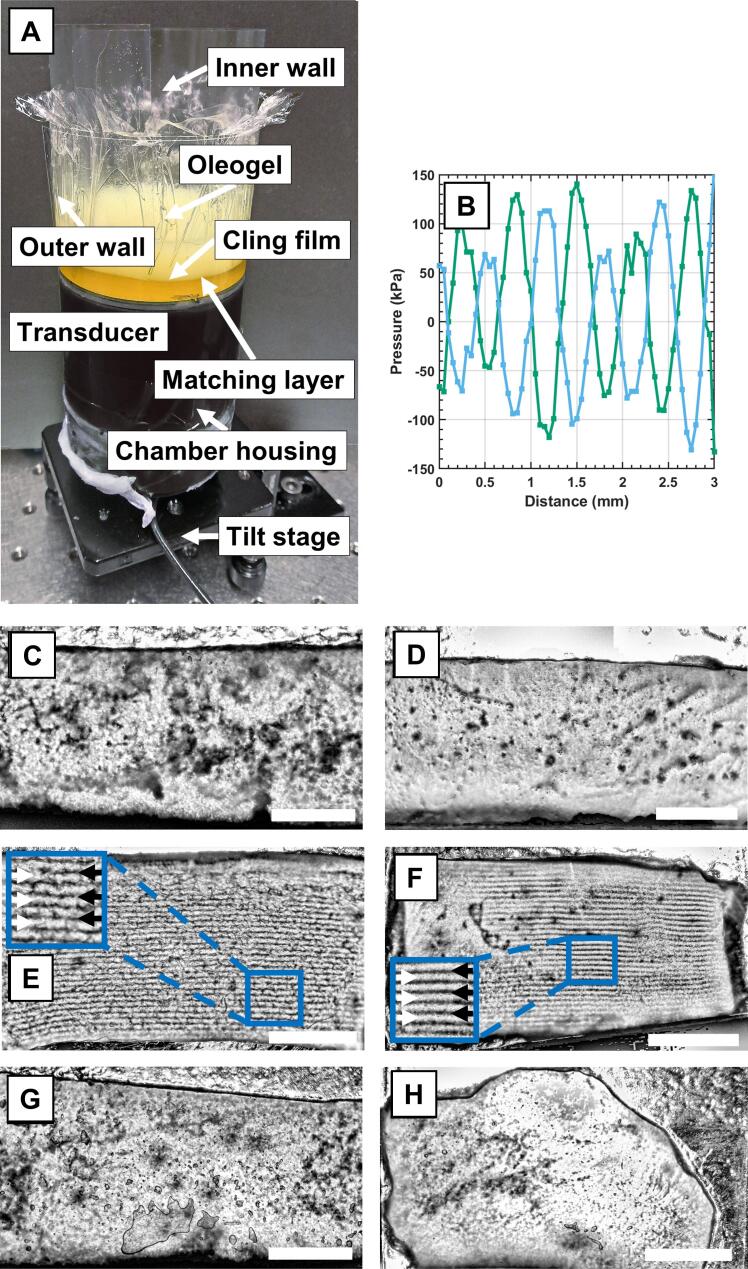
(A) Third design of the experimental chamber featuring a matching layer of oil between the transducer and the oleogel. Oleogel and matching layer were separated by cling film. (B) Pressure field scan recorded in oil during sonication in chamber three, 2.3 MHz; x-axis represents the distance from the cling film. Optical images of (C) 10% monoglyceride-containing (MG) sample reference, (D) 5% MG reference, (E) 10% MG medium-pressure sonicated, (F) 5% MG medium-pressure sonicated, (G) 10% MG low-pressure sonicated, and (H) 10% MG high-pressure sonicated sample slices. (C–H) Size bars equal 5 mm.

To solve the problem related to near-field effects, we developed a third chamber design (Fig. 2A) and introduced a matching layer between the transducer and sample. We selected oil as the matching layer because of its similar acoustic properties (sound speed) compared to molten oleogels. Using oil as the matching layer allowed for consistent coupling between the transducer and oleogel throughout the whole crystallization process. The oleogel and matching layer were separated using cling film. With this solution, the oleogel was relieved from any near-field effects. Since we redesigned the chamber (Fig. 2A), we first performed FEM simulations.

We simulated the new chamber geometry with flat cling film, which was parallel to the transducer and separated the matching layer and crystallizing oleogel. In addition, similar surface oscillations as those shown in Fig. 1C and Fig. 1D were studied. We observed that a time-averaged standing wave can be formed (Fig. S4A). Similar results were obtained if the separating cling film is convex, tilted, or wrinkled (Fig. S4B to Fig. S4D). To avoid problems during the extraction of the oleogel, we used a double-wall structure made of plastic film that ensured the stretching and proper positioning of the cling film (Fig. 2A). This was done even though simulations highlighted that the shape of the cling film does not affect the formation of the standing field. Subsequently, the presence of the standing wave was confirmed by vertically scanning the pressure profile of oil under sonication using a needle hydrophone (Fig. 2B). Effective actuation of the standing wave was visually verified using a dispersion of carbon rods in oil, which showed band-like structures as previously observed in both the first and second chambers (data not shown).

Then, 10% MG oleogels were sonicated during crystallization and a reference sample was produced with the same procedure without applying sonication. We selected three different sonication parameters that generated root-mean-squared pressures of 50 kPa, 70–110 kPa, and 180–240 kPa (hereafter called low, medium, and high, respectively), as determined by needle hydrophone measurements. After annealing, the oleogels were easily extracted from the chamber and sectioned. Fig. 2C–H show transversal sections of all samples. The samples sonicated at high and low pressures showed similar internal structures compared to the reference sample (cf. Fig. 2C with Fig. 2G and H). On the other hand, medium-pressure sonication (Fig. 2E) revealed a similar crystalline band-like structure as that observed in Fig. 1F (black arrows, second chamber design). In this case, the crystalline structure developed throughout the whole sample thickness, confirming the lack of near-field effects in the sample volume. By lowering the concentration to 5% MG, ease of sample extraction and band-like structure formation was maintained (*cf*. Fig. 2D and Fig. 2F). The third chamber was successful in producing extractable sonicated oleogels. We subsequently characterized the oleogels obtained in the last design for mechanical properties and oil release.

### 3.2 Mechanical properties and oil release

**Fig. 3:**
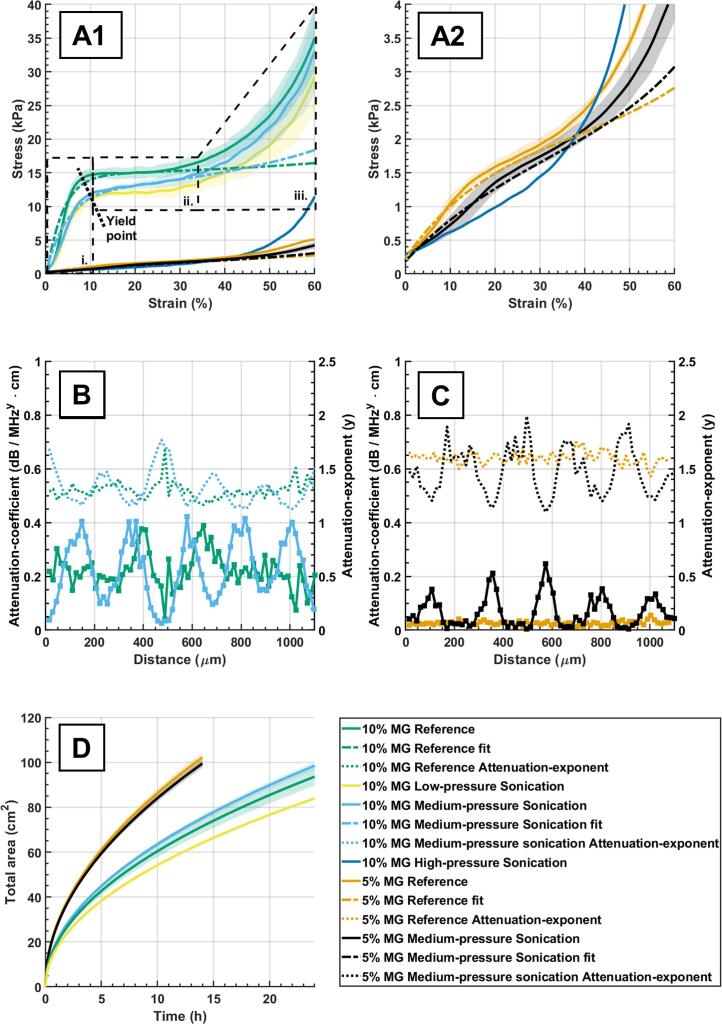
(A1) uniaxial-compression stress-strain curves for 10% and 5% monoglyceride-containing (MG) sample reference, 10% MG low-pressure sonicated, 10% and 5% MG medium-pressure sonicated, and 10% MG high-pressure sonicated samples. (A2) magnification of A1 showing uniaxial-compression stress-strain curves for 5% MG reference, 5% MG medium-pressure sonicated samples, and 10% MG high-pressure samples. (B and C) Local attenuation coefficients for 10% and 5% MG reference and 10% and 5% MG medium-pressure sonicated samples obtained from ultrasonic scanning microscopy imaging. Square dots are data points, while lines between dots are linear interpolations provided for easier observation of the trend. (D) Oil release for 10% and 5% MG reference, 10% and 5% MG medium-pressure sonicated, and 10% MG low-pressure sonicated samples. Shaded area represents 95% confidence interval in all plots.

We studied the mechanical properties of the oleogels obtained with the third chamber design. Fig. 3A1 shows uniaxial compression stress-strain curves for 10% and 5% MG reference and sonicated oleogels (force applied normal to the orientation of the planar band-like structures). Fig. 3A2 shows a magnification for the low-stress area. The effect of three different sonication pressure amplitudes (low, medium, and high) on 10% MG oleogel mechanical properties were also studied. All stress-strain curves for 10% MG samples were S-shaped, except for the high pressure-amplitude sonicated sample (Fig. 3A). These curves are typical for elastic-plastic foams[47], where the following three regions can be observed: the elastic region (Fig. 3A1 – i.), plateau region (Fig. 3A1 – ii.), and the densification region (Fig. 3A1 – iii.). From the elastic region, we calculated the effective stiffness for reference, low-, and medium-pressure sonicated samples, which were 2.78 ± 0.28 MPa, 1.93 ± 0.18 MPa, and 1.87 ± 0.15 MPa, respectively. Treated oleogels showed a shift in the yield point, *i.e.,* the point where the plateau region begins, to higher strain (shift to the right), except for the high-pressure sample (Fig. 3A1). Plateau and densification regions were higher for the reference samples compared to the sonicated samples, implying that sonicated samples can be deformed with less force and are therefore weaker compared to reference samples. On the other hand, high-pressure sonication formed the weakest oleogels as observed by the vertical shift of the strain-stress curve towards lower stresses (Fig. 3A1 and A2). For oleogels containing 5% MG, the curve shape for sonicated and reference samples lacked the plateau region and the lower amount of structuring agent led to a softer oleogel (Fig. 3A1 and A2). As observed for 10% MG samples, sonication shifted the curve below the reference sample.

In general, sonication during crystal formation affects the crystal length, interconnection among crystals, and crystal network structure[25], [48], [49]. This structural modification can change the mechanical properties of oleogels[25] (Fig. 3A1 and A2). This was observed when comparing reference sample with sonicated ones, keeping MG concentration constant. The formation of crystalline bands in the medium-pressure sonication did not change the overall mechanical properties compared to the low-pressure sonicated oleogels, characterized by the absence of a band-like crystalline structure. This could be because the dense MG crystal bands (Fig. 2E-F, black arrows) are visually thinner compared to the sparse MG crystal bands (Fig. 2E-F, white arrows). On the other hand, streaming, generated during high-pressure sonication, disturbed the formation of a proper crystalline network, which led to a softer material (Fig. 2H, A1 and A2). Moreover, it is also important to note that piezoelectric ceramics can generate heat during operation. This can impact the cooling-rate profile of the treated materials, as observed in our case (Fig. S2C), and possibly contributes to oleogel structuration and mechanical properties[50].

We then fitted the stress-strain curves using the material models of Ogden-Hill-Storåker (Storåker’s), Ogden, Yeoh, Blatz-Ko, and Fung. Storåker’s and Ogden models for foam-like materials were considered, as the experimental results showed stress-strain curves characteristic of elastic-plastic materials. Yeoh, Blatz-Ko, and Fung material models were considered, as they are general models for compressible polymers and biological tissue materials. Out of the five models, only Storåker’s (hyperelastic) model properly fitted the experimental data. To simplify the model (Eq. (2)), we first minimized the terms by calculating the Akaike information criterion (AIC) for *k* = 1, 2, and 3 for 5% and 10% reference samples. AIC scores for the three fits of 5% reference samples were 60.18, 66.18 and 72.22 at increasing *k*. Similar results were obtained for the 10% reference sample. The results thus indicate that Storåker’s model with *k* = 1 should be used to minimize the information loss. We assume that Storåker’s material model only describes the behaviour of the cellular structure in the oleogel. As such, when the oleogel is considerably deformed, and the cellular structure is destroyed and mixed with the oil fraction, we believe that Storåker’s model is no longer an accurate representation of the material. This means that the model is only applicable at the beginning of the compression, where the harder cellular structure dominates the observed stiffness.

Table 1 shows the results of the fitting. As expected, the fitted shear modulus increased with increasing MG concentration and was smaller for sonicated samples compared to their reference counterpart. A similar trend was observed for the α parameter. On the other hand, the β parameter was negative for the 10% MG samples. From this parameter, we calculated Poisson’s ratio (Eq. (3)), which was in the interval [-0.0322, −0.029] for 10% MG samples. The negative value of the ratio indicates that the material compresses in the perpendicular direction compared to the applied force, which does not correlate with the experimental observations. Indeed, oleogels have a positive Poisson’s ratio as experimentally observed. To validate the fitting parameters, we performed FEM simulations, which confirmed the validity of the model and fitted parameters (Fig. S5, supplementary material).

**Table 1:** Storåker’s model parameters obtained from fitting of uniaxial compression stress–strain curves (Fig. 3A1 and A2).

	10% MG Reference	10% MG Sonicated	5% MG Reference	5% MG Sonicated
Shear modulus (MPa)	2.1 ± 0.2*^,a^	1.3 ± 0.12*^,b^	0.006 ± 0.002*^,a^	0.004 ± 0.002*^,a^
α parameter	28.5 ± 3.2*^,a^	22.7 ± 2.6*^,b^	7.4 ± 6.3^ns,a^	6.1 ± 10.3^ns,a^
β parameter	−0.029 ± 0.002*^,a^	−0.022 ± 0.002*^,b^	−0.050 ± 0.023*^,a^	−0.017 ± 0.030^ns,a^
Poisson’s ratio	−0.031 ± 0.002*^,a^	−0.023 ± 0.003*^,b^	−0.057 ± 0.029*^,a^	−0.017 ± 0.033^ns,a^
R^2^	0.949	0.96	0.978	0.94

Note: * and ^ns^ refer to the significance of each parameter in the model for each sample, * = p < 0.05; ^ns^ = not significant, p > 0.05 (the parameter is not statistically different from 0).

^a,b^ = parameters with different letters within the same MG concentration are statistically different (p < 0.05).

Error represents the standard error of fitting.

The application of this model provided some insights into the internal structure of oleogels. This model predicts the behaviour of cellular foam-like materials under compression and properly describes the shape of the stress-strain curves of 10% MG oleogels up to 35% strain. Therefore, the mechanical behaviour of 10% MG oleogels can be modelled as a hyperelastic material and the oleogel structure can be idealized as a foam, where interconnected cellular elements are composed of MG platelet-like microcrystals and oil fills the whole structure. This is consistent with the most common crystalline network observed in oleogels containing fatty alcohols[15] and vegetable waxes[51], [52]. The estimated parameters for the 5% MG samples were statistically insignificant. This can be attributed to the shape of the experimental stress-strain curves, which do not resemble the stress-strain curves of hyperelastic foams.

To better understand the role of the oleogels band-like microstructure (Fig. 2E and F) on the mechanical behaviour of the sample, we measured frequency-dependent local acoustic attenuation by scanning the sample with a Scanning Acoustic Microscope (SAM). The obtained data were fitted to an empirical frequency power law (Eq. (5)), from which we calculated the attenuation coefficient and the frequency-dependent exponent. These parameters correlate with the internal structure and viscoelasticity of viscoelastic materials[53]. Fig. 3B and C show the acoustic attenuation coefficient (*α_0_*) and exponent (*y*) as a function of scanning distance for 10% and 5% MG reference and sonicated samples (medium pressure). The figures show peaks and troughs for both sonicated samples, whereas the reference samples show an irregular change in the attenuation coefficient across the sample length. We observed a similar behaviour in the attenuation exponents, except that they were in antiphase compared to the attenuation coefficients. In addition, the attenuation coefficients for 5% MG samples were lower than those of 10% MG oleogels, while the exponent showed an opposite trend. The 5% MG reference showed less variation in the attenuation coefficient and exponent across the sample length when compared with the 10% MG reference.

Acoustic attenuation in inhomogeneous media may be due to two distinct simultaneous independent linear phenomena, namely scattering and viscous losses[50]. The results show that an increase in MG led to a lower y and a higher *α_0_,* which in combination results in a higher overall attenuation in the frequency range used (see Eq. (5)). The increase in attenuation for the higher MG concentration oleogels results in a larger loss tangent (Eq. (6)), which would indicate that an increase in gelator concentration results in a more liquid material. This contradicts the known relationship between gelator concentration and material behaviour of oleogels[19], [25], [54]. Therefore, the attenuation observed in Fig. 3B and 3C is likely due to scattering. This is plausible because in our case there was a difference in acoustic impedance between the solid MG crystals and liquid oil, and the wavelength used during SAM measurements in oil (44–166 µm) is comparable to the size of MG microcrystals (usually in the 30–100 µm range[25], [43]), which gives rise to Mie scattering[50]. Based on these observations, the peaks in *α_0_* and troughs in *y* for sonicated oleogels (Fig. 3B, 3C) are due to higher scattering of the propagating wave and can indicate a local accumulation of MG crystals, which correspond to the crystalline structures optically observed in Fig. 2E and F. This means that MG bands are mechanically different compared to the surrounding material due to their local increased crystal concentration. This then leads to an increase in the local solid-like behaviour of the material. The opposite also holds, where areas with low concentration of MG have a more viscous-like behaviour and are located between the denser regions. This can explain the lower uniaxial compression curve for sonicated samples (Fig. 3A1). Indeed, low MG concentration areas are compressed before the high MG concentration areas, leading to an overall weakening of the oleogel structure.

Finally, to evaluate the effect of USW structural and mechanical modifications on oleogels, we characterized the oil-release profiles for the 10% and 5% MG medium-pressure sonicated samples, 10% MG low-pressure sonicated sample, and 10% and 5% MG reference samples. Fig. 3D shows the evolution of the oil stain area on filter paper as a function of time. All samples showed a similar oil-release profile, where 5% MG samples had a faster oil release compared to the 10% MG samples. No visible differences were noted between the medium-pressure sonicated and reference samples within the same MG concentration. To gain further insight into the sonication effect on oleogels, we fitted an exponential equation (Eq. (7)) to the data and used an average value of 0.5 for the exponent *n*, thus allowing us to compare the oil-release-rate constant (*K*) values among samples. The 5% MG reference and sonicated samples had a *K* value of 26.6 ± 1.1 and 27.3 ± 1.1, respectively. The 10% MG reference, low-, and medium-pressure sonicated samples had a *K* value of 19.1 ± 1.0, 17.1 ± 0.5, and 20.1 ± 0.5, respectively. Sonication modified the oil release compared to the reference. For low-pressure sonicated samples, the oil release was slower compared to the reference sample. Medium-pressure sonication and reference 10% MG samples showed similar behaviour, whereas the difference between 5% MG reference and medium-pressure sonicated samples was statistically insignificant. The weakening of the overall oleogel structure for medium-pressure sonicated samples, as suggested by the uniaxial compression test, seems to hasten oil release. In addition, the formation of band-like structures does not seem to have affected oil release. This behaviour contradicts our previous results from a colourant migration test[28]. It should be noted that sample manipulation could have affected the surface in contact with the filter paper, leading to similar oil-release results between sonicated and reference samples that have the same MG concentration. In addition, oil release tested with filter paper (this work) and colourant migration results[28] should not be compared, as they use capillary (oil pulled out from the oleogel structure) and diffusive (colorant enters in the oleogel structure) mechanisms, respectively.

## 4 Conclusions

We designed and developed an USW chamber with a 10–100 mL treatable volume. The chamber design was refined over three different variants, leading to a final design that allowed for the extraction of sonicated samples with minimal to no damage. Medium-pressure sonicated samples showed optically observable band-like structures throughout the sample. These bands were mechanically different compared to the surrounding material as verified by SAM and were responsible for the bulk mechanical behaviour of treated oleogels. By fitting the stress-strain data with Storåker’s material model, we highlighted the fact that high concentrations of gelators (*i.e.,* 10% MG) can lead to a mechanical behaviour resembling that of a hyperelastic foam. Finally, by employing USW we modified the oil-release profiles of oleogels. A summary of the effect of USWs on oleogel microstructure, bulk and local mechanical properties, and oil release compared to untreated oleogel are shown in Table 2.

**Table 2:** Composite table of changes caused by sonication.

	**Reference Oleogels**	**Treated Oleogels**
**Microstructure**	Irregular	Band-like (100 ± 50 µm thick) only for medium-pressure treated oleogels
**Bulk Mechanical Properties**		
*Effective Stiffness*	−	
*Plateau and Densification*	−	
*Yield Point*	−	Higher strain
*Stress-Strain Curve Shape*	Foam Like/Linear	Unchanged/Exponential
**Local Mechanical Properties**		
*Attenuation Coefficient*	Constant/High Variance	▲/Periodical
*Attenuation Exponent*	Constant/High Variance	▾/Periodical
**Oil Release**		

Arrows indicate a relative change in the parameter in question. Plus signs in the oil release indicate a relative amount of released oil. Colour codes indicate if the change is desirable (orange: undesirable, blue: desirable).

The developed practical method may perhaps allow local control of oleogel structure and mechanical properties, which is a step towards a possible application of USWs in the food, pharmaceutical, and cosmetic industries.

### CRediT authorship contribution statement

**Petri Lassila:** Methodology, Software, Formal analysis, Investigation, Data curation, Writing – original draft, Writing – review & editing, Visualization. **Fabio Valoppi:** Conceptualization, Methodology, Formal analysis, Investigation, Resources, Writing – original draft, Writing – review & editing, Visualization, Project administration, Funding acquisition. **Oskari Tommiska:** Methodology, Software, Formal analysis, Investigation, Data curation, Writing – review & editing. **Jere Hyvönen:** Methodology, Software, Formal analysis, Investigation, Data curation, Writing – review & editing. **Axi Holmström:** Methodology, Writing – review & editing. **Sami Hietala:** Methodology, Resources, Writing – review & editing. **Ari Salmi:** Conceptualization, Methodology, Resources, Writing – review & editing, Project administration, Funding acquisition. **Edward Haeggström:** Resources, Writing – review & editing, Funding acquisition.

## Declaration of Competing Interest

The authors declare the following financial interests/personal relationships which may be considered as potential competing interests: Fabio Valoppi reports was provided by Horizon 2020. Fabio Valoppi has patent #US20210127720A1 pending to University of Helsinki. Ari Salmi has patent #US20210127720A1 pending to University of Helsinki. Edward Haeggstrom has patent #US20210127720A1 pending to University of Helsinki.
