# Supplementary figures and images for: Practical scale modification of oleogels by ultrasonic standing waves
Source: Ultrason Sonochem. 2022 Mar 3;85:105970. doi: 10.1016/j.ultsonch.2022.105970 (PMC8983462; doi:10.1016/j.ultsonch.2022.105970)

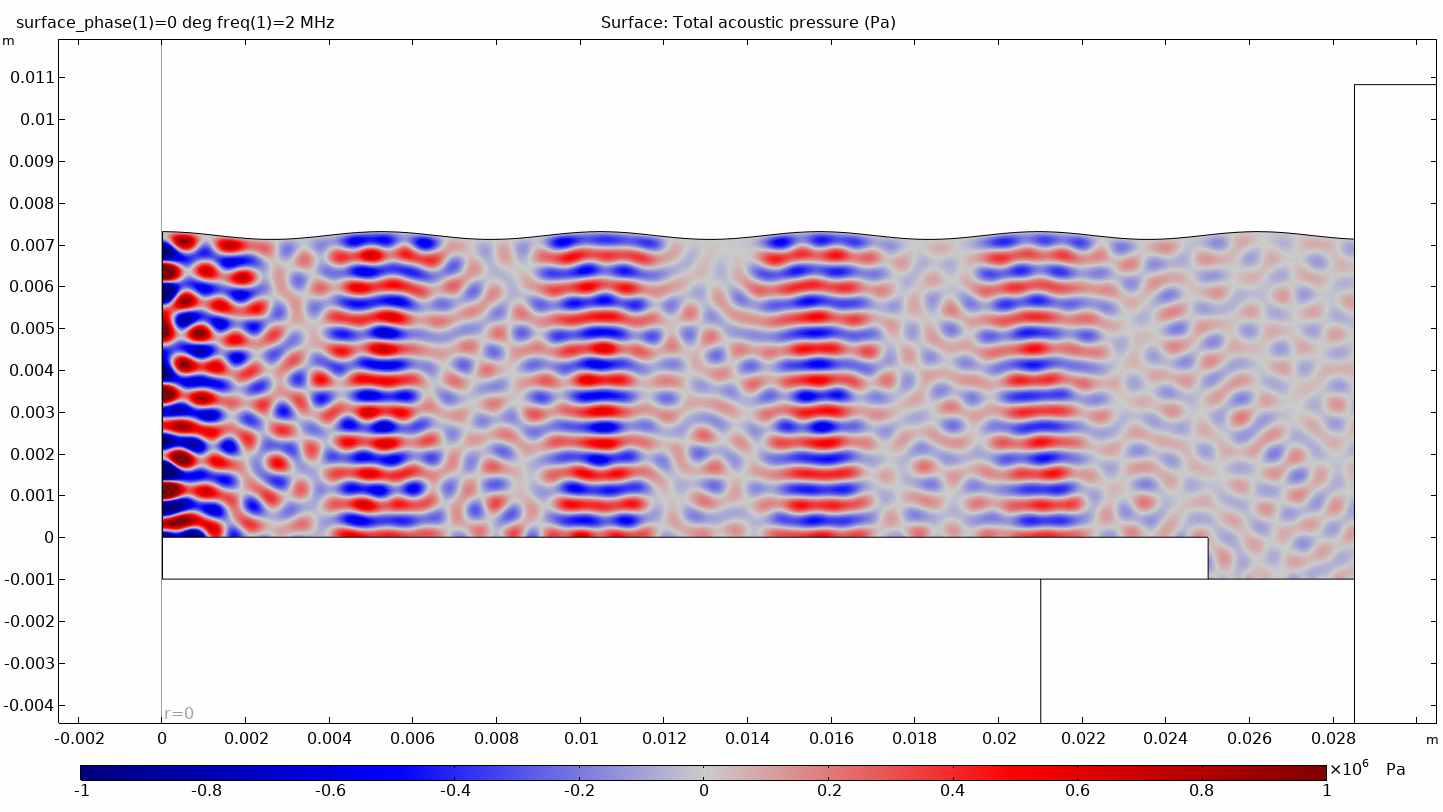

Supplement: Supplementary video 3 [file mmc3.gif]

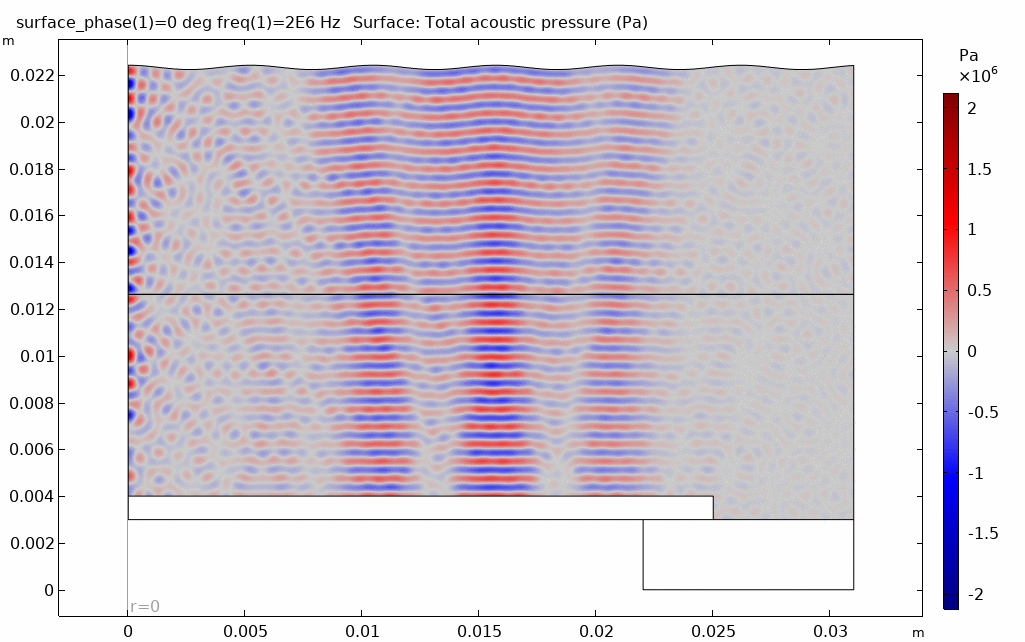

Supplement: Supplementary video 4 [file mmc4.gif]

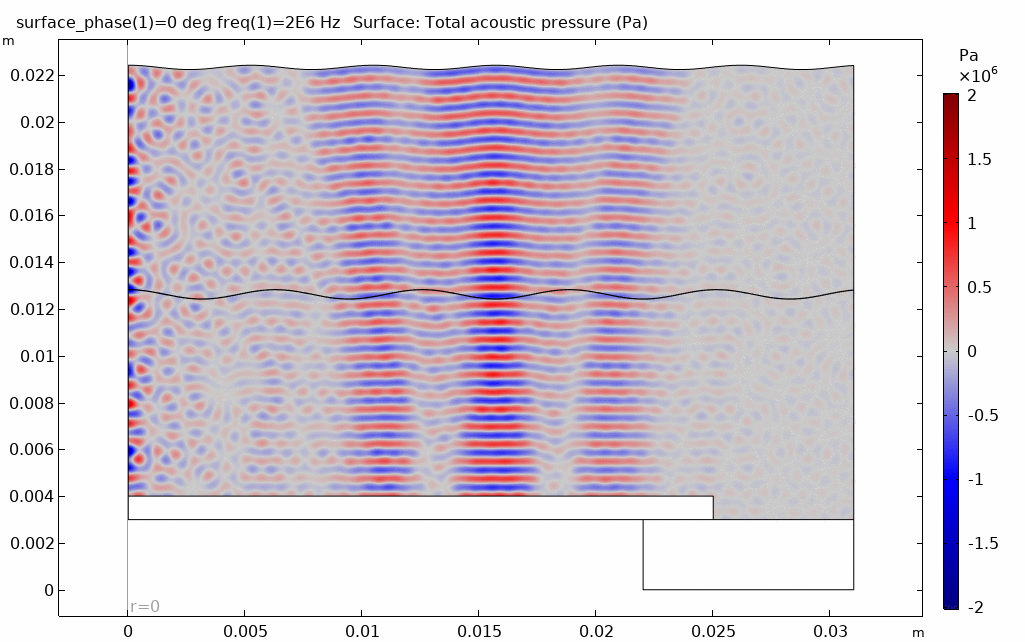

Supplement: Supplementary video 5 [file mmc5.gif]

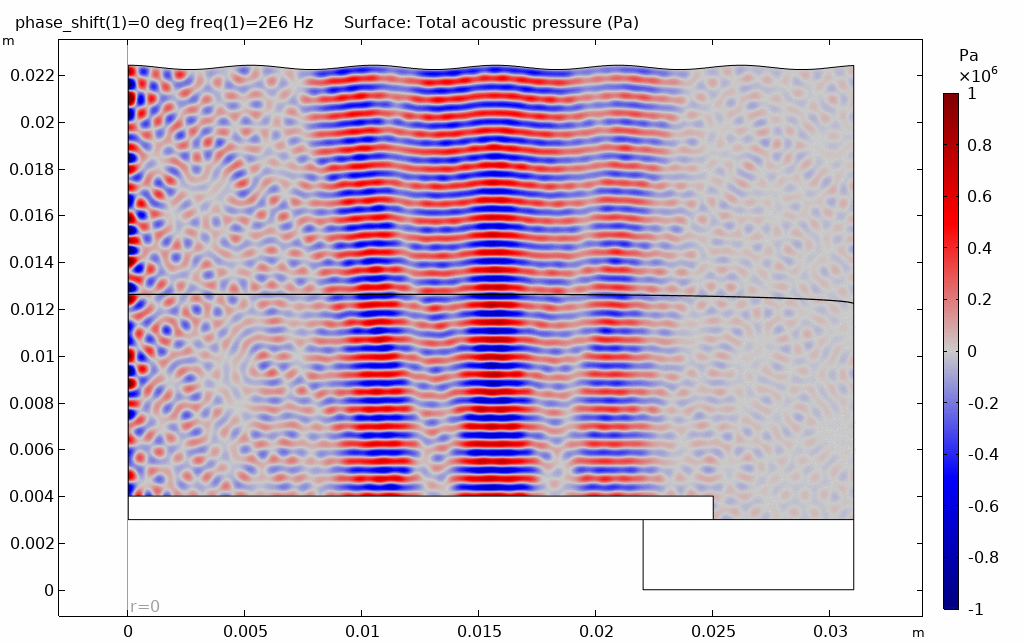

Supplement: Supplementary video 6 [file mmc6.gif]

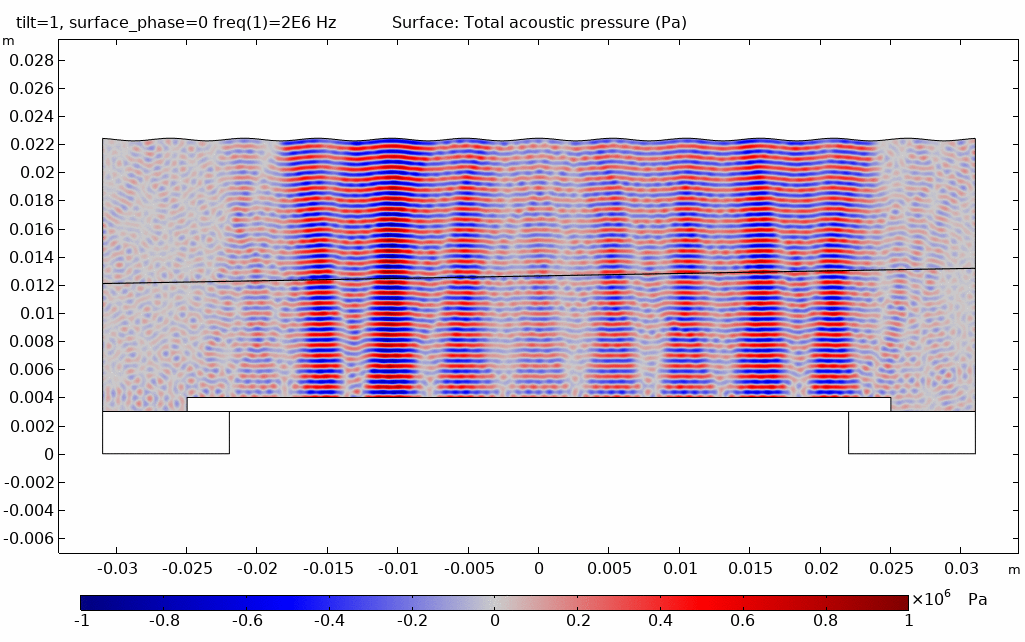

Supplement: Supplementary video 7 [file mmc7.gif]
