# Supplementary material for: Practical scale modification of oleogels by ultrasonic standing waves
Source: Ultrason Sonochem. 2022 Mar 3;85:105970. doi: 10.1016/j.ultsonch.2022.105970 (PMC8983462; doi:10.1016/j.ultsonch.2022.105970)
Supplement: Supplementary data 1 [file mmc1.docx]

SUPPLEMENTARY MATERIAL

Practical Scale Modification of Oleogels by Ultrasonic Standing Waves

Petri Lassila^1^, Fabio Valoppi^1,2,3,*^, Oskari Tommiska^1^, Jere Hyvönen^1^, Axi Holmström^1^, Sami Hietala^4^, Ari Salmi^1^, Edward Haeggström^1^

Affiliations:

^1^ Electronics Research Laboratory, Department of Physics, P.O. Box 64 (Gustaf Hällströmin katu 2), FI-00014 University of Helsinki, Finland

^2^ Department of Food and Nutrition, P.O. Box 66 (Agnes Sjöbergin katu 2), FI-00014 University of Helsinki, Finland

^3^ Helsinki Institute of Sustainability Science, Faculty of Agriculture and Forestry, FI-00014 University of Helsinki, Finland

^4^ Department of Chemistry, University of Helsinki, P.O. Box 55 (Virtasen aukio 1), FI-00014 University of Helsinki, Finland

*Corresponding author: fabio.valoppi@helsinki.fi


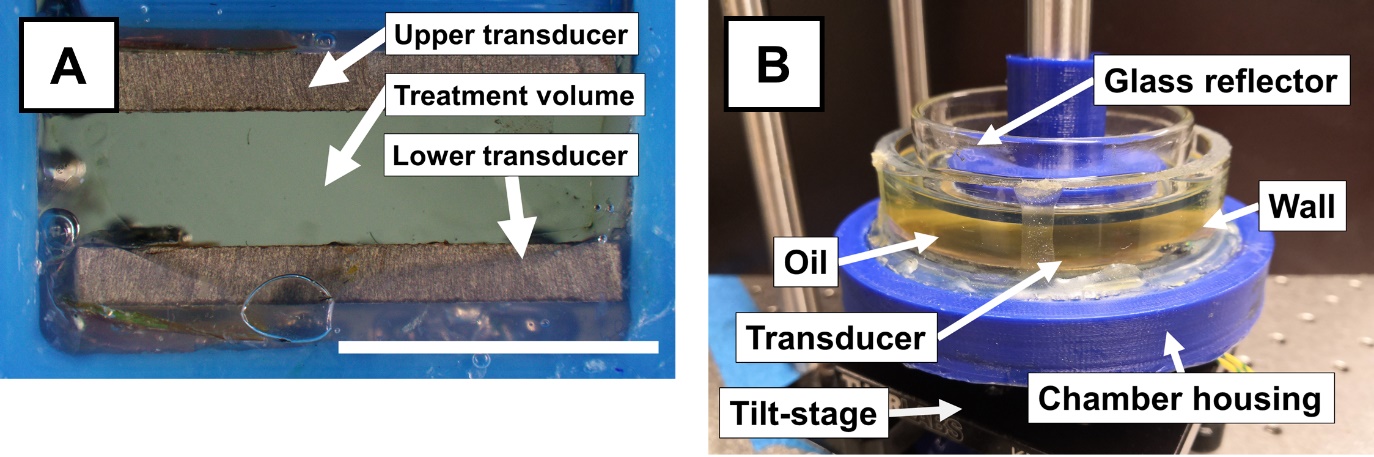


**Fig. S1** (A) Microfluidic treatment chamber previously developed; size bar represents 5 mm. (B) First design of the scaled-up chamber. Size bar represents 10 mm.


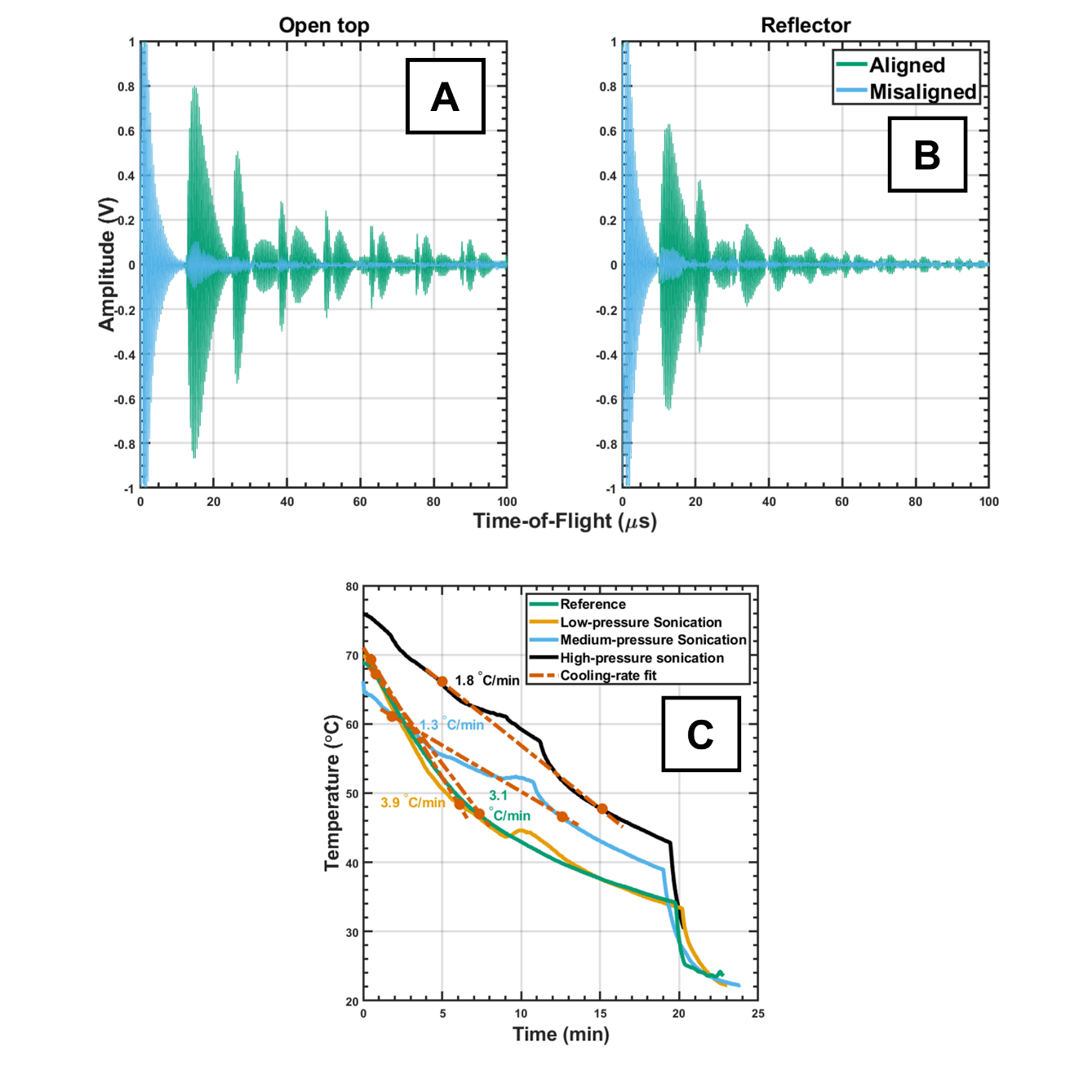


**Fig. S2** (A) Pulse-echo measurements for aligned and misaligned open-top (oil-air interface) chamber. (B) Pulse-echo measurements for aligned and misaligned closed-top (glass reflector) chamber. (C) Temperature measurement over time for crystallizing 10% MG oleogels. The cooling rate for reference oleogels was 3.1 ºC/min, for low-pressure sonication oleogels 3.9 ºC/min, for medium-pressure sonication oleogels 1.3 ºC/min, and for high-pressure sonication oleogels 1.8 ºC/min.

**Near-field effect calculation**

$N=\frac{D^{2}f}{4c}$

Where *N* is the near-field distance, *D* is the diameter of the piezoceramic transducer, *f* is frequency, and *c* is the sound velocity in sonicated medium (oil). This was calculated for a 50-mm transducer, immersed in oil (*c* = 1480 m/s), sonicated at 2.25 MHz, which for the near-field distance was approximately 1 mm.


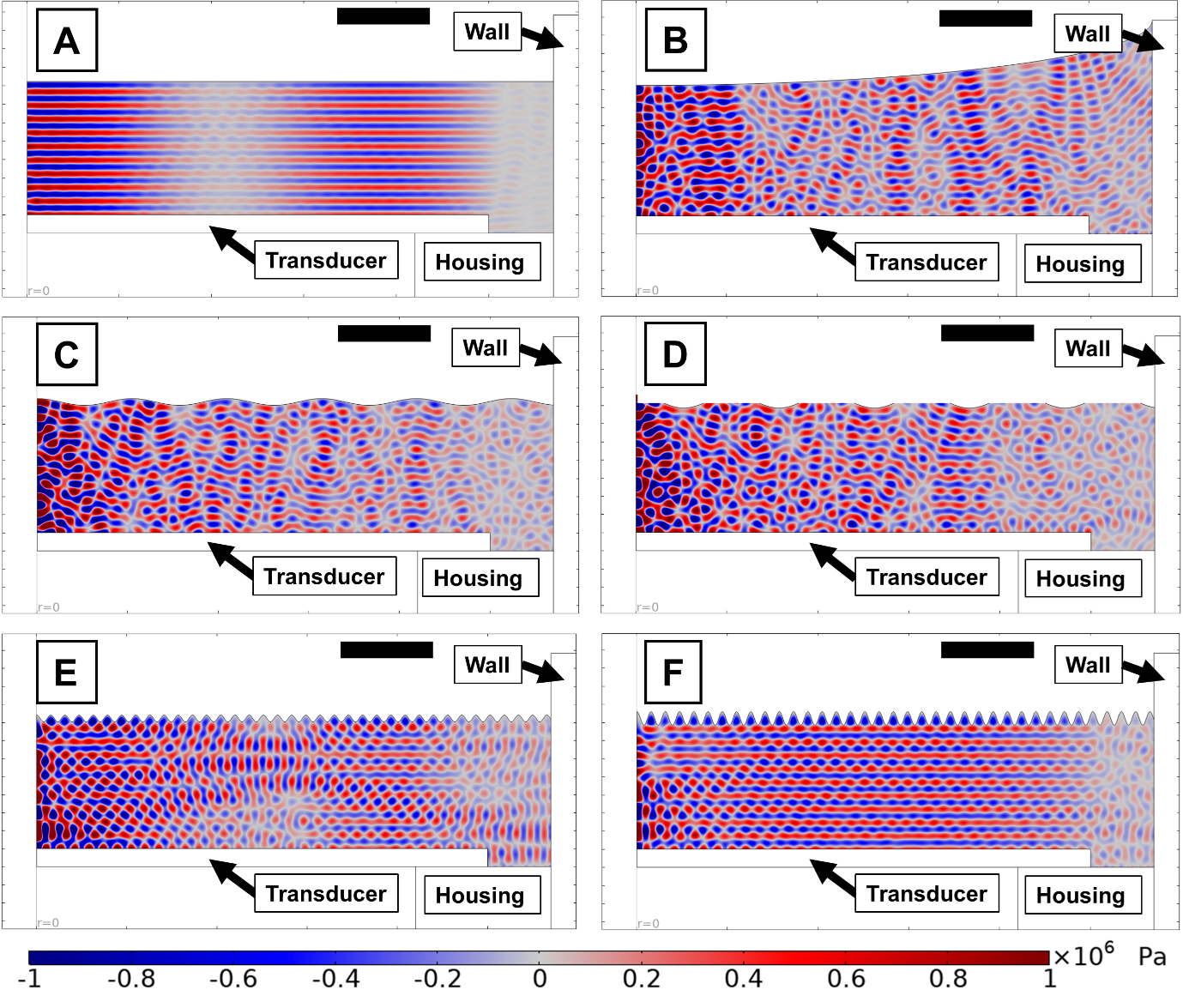


**Fig. S3** Open-top (oil-air interface) pressure field simulations for chamber 2 with (A) flat surface, (B) major meniscus affecting the whole sonication area, (C) rippled surface, with surface-amplitude λ/2 and spatial-frequency 1.2 mm^-1^, (D) rippled surface, with surface-amplitude λ and spatial-frequency 1.2 mm^-1^, (E) rippled surface, with surface-amplitude λ/2 and spatial-frequency 0.2 mm^-1^, and (F) rippled surface, with surface-amplitude λ and spatial-frequency 0.2 mm^-1^.


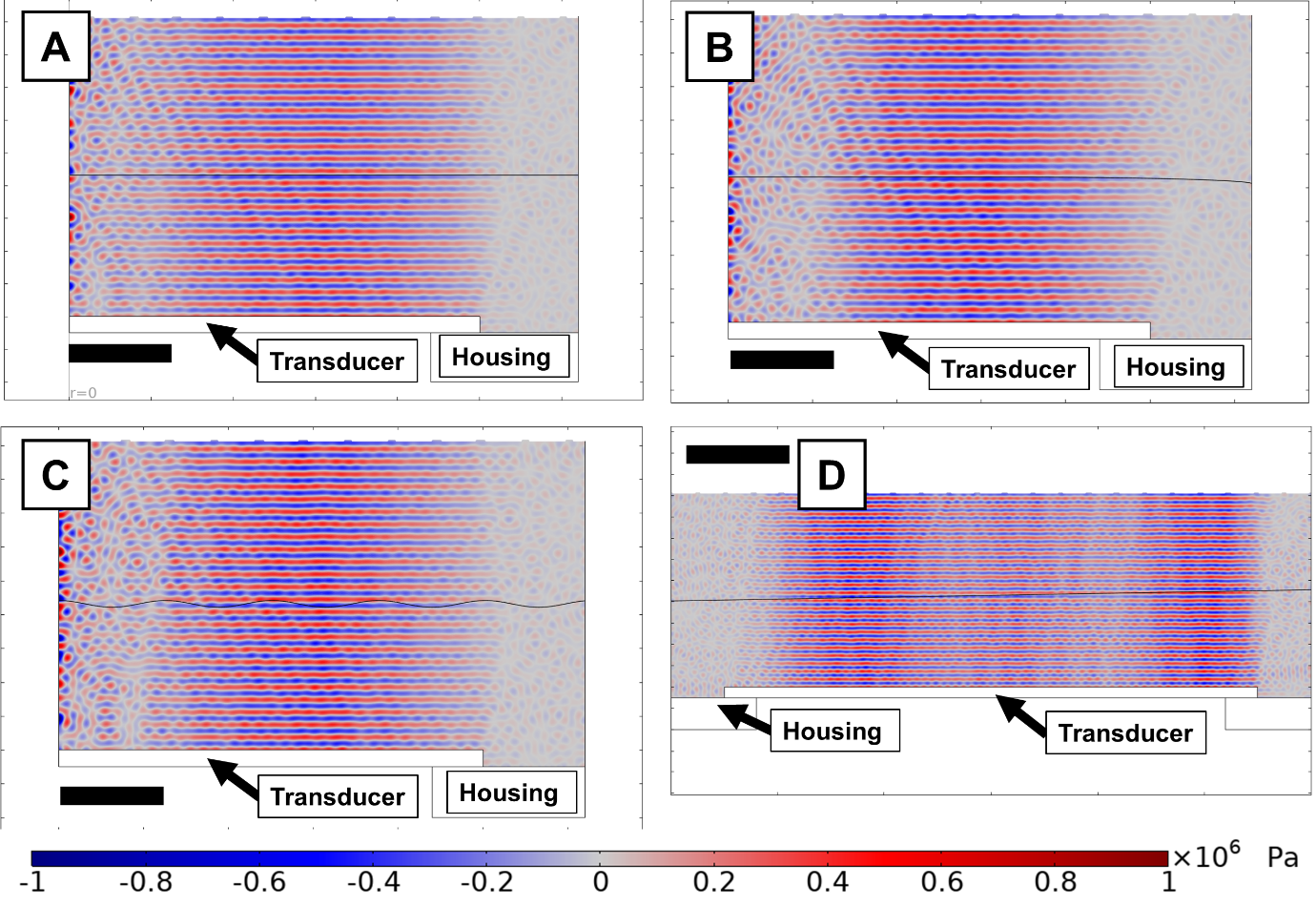


**Fig. S4** Time-averaged open-top (oil-air interface) pressure field simulations for chamber 3 with (A) flat cling film layer in between, (B) cling film layer bent on the edges, (C) rippled cling film layer, (D) tilted cling film layer.


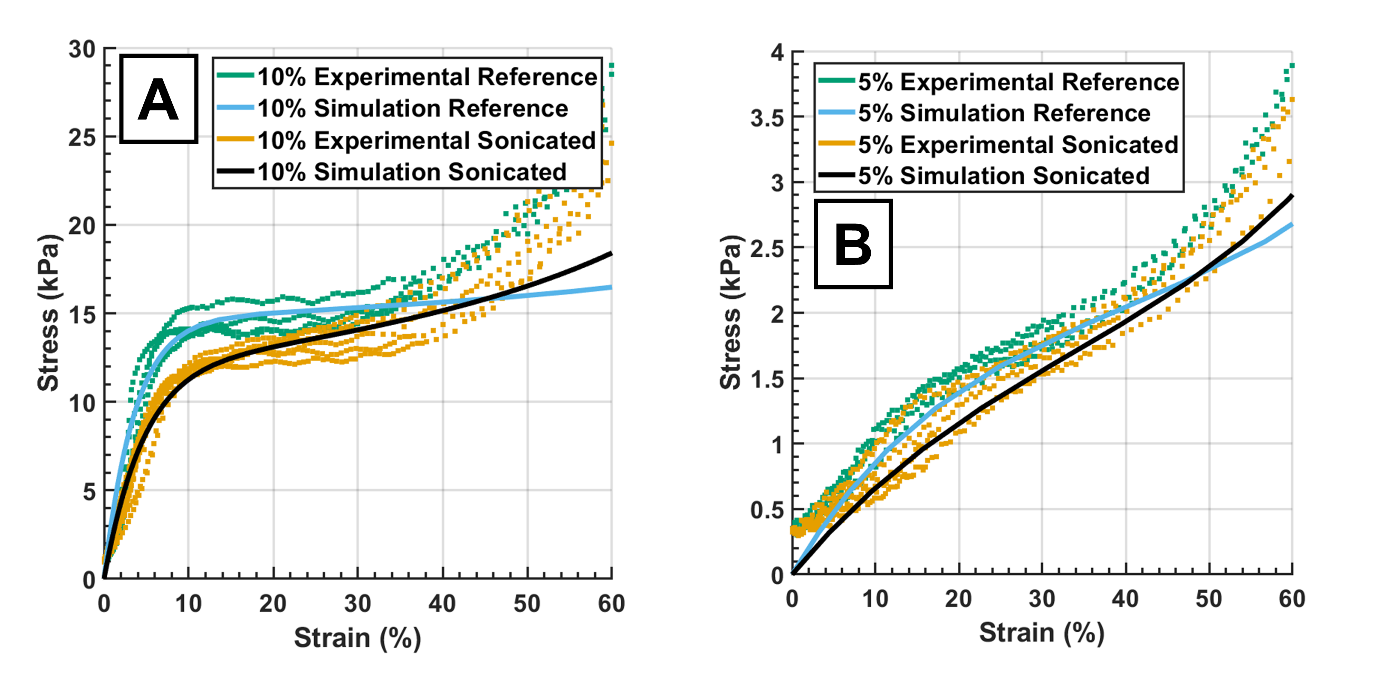


**Fig. S5** Uniaxial-compression simulations with parameters derived from Storåker’s model for (A) 10% MG medium sonicated and reference samples and (B) 5% MG medium sonicated and reference samples.


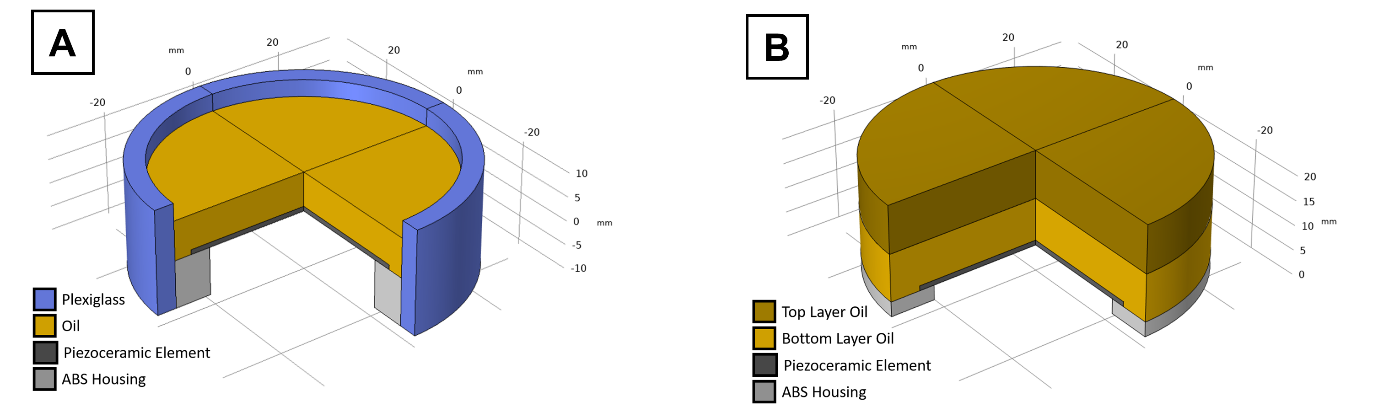


**Fig. S6** (A) Simulation geometry used in pressure-field simulations for chamber design 2. (B) Simulation geometry used in pressure-field simulations for chamber design 3.

**Captions for supplementary GIF and MP4 files**

**Case-2** Frequency domain, changing reflective surface open-top (oil-air interface) pressure field simulations for chamber 2.

**Case-3** Frequency domain, changing reflective surface open-top (oil-air interface) pressure field simulations for chamber 3 with (A) flat cling film layer in between, (B) cling film layer bent on the edges, (C) rippled cling film layer, (D) tilted cling film layer.

**Video-1** Video of 30 mL of rapeseed oil under high-pressure sonication.
